# Marginal Bone Changes Around Tissue‐Level Implants After Prosthesis Delivery: A Multicenter Prospective Study

**DOI:** 10.1111/cid.70071

**Published:** 2025-06-11

**Authors:** Sergio Spinato, Fabio Bernardello, Claudio Stacchi, Carlo Maria Soardi, Marcello Messina, Antonio Rapani, Teresa Lombardi

**Affiliations:** ^1^ Private Practice Sassuolo Italy; ^2^ Private Practice Legnago Italy; ^3^ Department of Medical, Surgical and Health Sciences University of Trieste Trieste Italy; ^4^ Private Practice Brescia Italy; ^5^ Private Practice Trieste Italy; ^6^ Department of Medical and Surgery Specialties, Radiological Sciences and Public Health, Dental School University of Brescia Brescia Italy

**Keywords:** bone remodeling, crown emergence profile, early marginal bone loss, mucosal thickness, prosthetic loading, tissue‐level implants, transmucosal collar height

## Abstract

**Introduction:**

Early marginal bone loss (EMBL) is a non‐infective phenomenon occurring around the implant neck from placement to the first year of prosthetic function, being influenced by both surgical and prosthetic factors. This multi‐center prospective study assesses the impact of different variables potentially influencing marginal bone stability during the period from crown delivery to 18 months of functional loading.

**Methods:**

Forty‐seven patients requiring a single posterior mandibular implant were selected according to specific criteria. Tissue‐level implants were placed at different crestal bone levels based on vertical mucosal thickness and followed in an unsubmerged healing protocol, as described in a previous study evaluating peri‐implant bone levels (PBL) from implant placement (T0) to crown delivery (T1). The present study continues this evaluation, focusing on MBL from T1 to 18 months post‐loading (T2). Host‐related factors (age, gender, smoking, history of periodontitis, vertical mucosal thickness) and specific prosthetic parameters, including crown mesio‐distal dimension, emergence angle, and transmucosal collar height were recorded. Multiple linear regression analysis explored associations between MBL and prosthetic or patient‐related factors, with significance set at *p* < 0.05.

**Results:**

Marginal bone levels remained stable from T1 to T2, with no significant association between MBL and host‐related factors or defined prosthetic variables. However, total MBL from T0 to T2 was significantly higher around implants with thin mucosa at T0 compared to medium and thick mucosa. Multivariate analysis (T0‐T2) identified thin mucosa and smoking as significant MBL predictors.

**Conclusion:**

From T1 to T2, marginal bone levels around tissue‐level implants remain stable, with no significant influence from variables analyzed. Conversely, peri‐implant bone resorption between T0 and T2 is significantly associated with thin mucosa (primarily affecting T0‐T1) and smoking.

**Trial Registration:** www.clinicaltrials.gov: NCT05363306

## 1 Introduction

Early marginal bone loss (EMBL) is a common, noninfectious, and nonprogressive phenomenon that typically occurs around the implant neck from the time of placement through the first year of prosthetic function [1, 2]. Following this initial period, marginal bone levels are expected to remain relatively stable over time [3]. Consequently, the assessment of peri‐implant marginal bone stability is considered a key parameter in evaluating the long‐term success of dental implants [3, 4]. It was traditionally accepted that up to 2 mm of EMBL may occur in the first year after implant loading. However, this 2‐mm threshold defining implant success has been reconsidered by more recent investigations [5, 6]. Prospective long‐term studies highlight that implant survival, late peri‐implant bone loss (BL) and peri‐implantitis development are significantly influenced by EMBL ≥ 0.5 mm [7, 8].

EMBL has a multifactorial etiology and is influenced by a combination of implant‐related, patient‐related, surgical, and prosthetic factors [2, 9].

Surgical factors include insufficient bone crest width, thermal and mechanical trauma during implant site preparation, excessive cortical compression during implant insertion and implant crest module characteristics [2, 9, 10, 11, 12]. These factors can similarly affect EMBL around both bone‐level and tissue‐level implants during the surgical phase. In addition, supra‐crestal tissue height (STH) adhesion fundamentally conditions peri‐implant EMBL prior to prosthesis delivery and loading [2, 9, 13, 14]. When bone‐level or tissue‐level implants become exposed to the oral cavity, soft tissues form a “cuff‐like” barrier sealing the trans‐mucosal component. Tomasi and co‐workers showed that in humans the vertical dimension of this mucosal barrier around bone‐level implants ranges from 1.8 to 3.5 mm and is completely developed within 2 months [15]. In this phase of supra‐crestal tissue maturation, peri‐implant bone resorption may occur, especially when thin mucosa (≤ 2 mm) is present, as revealed in pre‐clinical [16, 17] and clinical studies [18, 19, 20]. If mucosal thickness is insufficient to establish a proper mucosal seal around the implant, peri‐implant marginal bone resorption occurs apically to allow adequate space for supra‐crestal tissue adhesion [16, 17].

Prosthetic factors may also significantly condition EMBL [2, 9, 10]. Several studies have demonstrated that the pattern of peri‐implant EMBL is time‐related after implant placement. The most significant EMBL around bone‐level implants placed using a two‐stage approach occurs after implant uncovering and final abutment connection, with a marked increase up to 6 months after prosthetic loading [5, 21, 22]. Therefore, multiple abutment connections/disconnections [23], abutment insertion timing [24, 25], prosthetic abutment height [21, 22, 26, 27, 28, 29], prosthetic abutment shape and emergence profile [30], and adaptive response to occlusal function [31] may influence further peri‐implant BL around bone‐level implants.

Emerging evidence indicates that EMBL around tissue‐level implants primarily occurs in the immediate post‐operative period, specifically during the soft tissue healing phase around the transmucosal portion, prior to the initiation of functional loading [18, 32]. Owing to the coronally displaced location of the implant–abutment micro‐gap in tissue‐level designs, it is biologically plausible that prosthetic factors exert a comparatively lower impact on EMBL than in bone‐level implants [33]. This hypothesis is indirectly supported by a cross‐sectional study showing that implant‐abutment emergence angle (EA) and profile are significant predictors of EMBL in bone‐level implants, whereas no such association was observed in tissue‐level fixtures [30].

However, to the best of the authors' knowledge, there is no strong evidence currently supporting a direct association between peri‐implant BL around tissue‐level implants and prosthetic design characteristics. This multicenter prospective study was therefore designed to investigate the factors that may influence marginal bone remodeling (BR) around tissue‐level implants over an 18‐month period following prosthesis delivery, including both prosthetic‐ and patient‐related variables.

## 2 Materials and Methods

### 2.1 Experimental Design

This multi‐center prospective study was reported following STROBE (Strengthening the Reporting of Observational studies in Epidemiology) guidelines. All procedures were in full accordance with the principles outlined in the WMA Helsinki Declaration and subsequent modifications (Fortaleza 2013) [34]. The study protocol was approved by the relevant Ethical Committee (Regione Calabria, Sezione Area Centro, Nr. 370/2020), and recorded in a public register of clinical trials. All selected patients signed written informed consent in which all clinical procedures, possible risks and therapeutic alternatives were detailed. Patients allowed use of their personal data for research purposes.

All the clinical centers participated in a calibration meeting prior to the clinical study to discuss study protocol and standardize collection of experimental parameters to obtain acceptable inter‐examiner consistency.

### 2.2 Patient Selection

This multi‐center prospective study is based on data from a set of patients, included in a previously published 5‐month clinical trial [20]. All patients, selected consecutively from a pool, were treated in six clinical centers. Patients were partially edentulous and required placement of at least one single implant in native bone in the posterior mandible. In the case of multiple implants, only the most mesial implant was evaluated such that each patient contributed to the study with only one implant.

General inclusion criteria were as follows:

– age > 18 years;
– good general health;
– absence of systemic disease affecting wound healing and bone metabolism;
– no regular medication consumption for at least 3 months prior to treatment;
– patient willingness and ability to fully comply with the study protocol;
– written informed consent given.

Local inclusion criteria were as follows:

– presence of keratinized mucosa at implant site with 3‐mm minimum buccolingual width;
– bone crest at implant site with a minimum of 6 mm width and 9 mm height above the mandibular canal, with no previous or concomitant bone augmentation procedure;
– healed bone crest (at least 6 months elapsed from tooth extraction/loss);
– presence of opposing dentition.

Additional exclusion criteria were as follows:

– history of head or neck radiation therapy;
– uncontrolled diabetes (HBA1c > 7.5%);
– active infection;
– immunocompromised patients (HIV infection or chemotherapy within the past 5 years);
– present or past treatment with intravenous antiresorptives;
– patient pregnancy or lactating at any time during the study;
– poor oral hygiene and motivation (full mouth plaque score FMPS > 25%);
– untreated periodontal disease;
– psychological or psychiatric problems;
– alcohol or drug abuse;
– participation in other studies, if the present protocol could not be correctly followed;
– peak insertion torque > 60 Ncm.

Patients received oral hygiene instruction and professional deplaquing 1 week prior to implant placement surgery.

### 2.3 Surgical and Prosthetic Procedures

Surgical procedures, described in detail in a previously published 5‐month clinical trial [20], are summarized as follows: under local anesthesia, a mid‐crestal incision along the center of the edentulous bone crest was carried out. A full‐thickness flap was elevated in two phases as described elsewhere [20].

1. after full thickness buccal flap elevation, a soft‐tissue probe (SSL, Medesy, Maniago, Italy) was used at the center of the future implant site to measure the vertical height of the un‐separated lingual flap;
2. full thickness lingual flap elevation was then performed, exposing the alveolar ridge.

A one‐stage surgical protocol was employed. Implant sites were prepared for the placement of tissue‐level implants characterized by a 3 mm‐high convergent, machined transmucosal collar, a moderately rough endosseous surface, and an external hexagonal connection (i‐Smart, i‐Res, Lugano, Switzerland). The convergent geometry of the transmucosal collar enabled individualized apico‐coronal positioning relative to mucosal thickness, thereby eliminating the need for countersinking and ensuring optimal soft tissue adaptation. Vertical mucosal thickness was assessed following buccal flap reflection, and implants were placed at three crestal levels accordingly: (Group 1) 2 mm subcrestal in sites with thin mucosa (< 2.5 mm); (Group 2) 1 mm subcrestal in sites with medium mucosa (2.5–3.5 mm); and (Group 3) equicrestal in sites with thick mucosa (> 3.5 mm).

Operators selected appropriate implant length (8 or 10 mm) according to available bone height. All implants were left unsubmerged and covered with a healing cap. The flaps were sutured around the transmucosal component, allowing soft tissue maturation to occur passively without the use of provisional crowns or additional soft tissue conditioning. Patients were prescribed antibiotic therapy for 6 days and non‐steroidal anti‐inflammatory drugs when needed. Sutures were removed 12–14 days after surgery. No removable prostheses were utilized by patients during the healing period.

After 5 months, patients were referred to prosthodontists for subsequent prosthetic rehabilitation.

The final impression was taken using a polyether material (Impregum, 3M, Milan, Italy). After functional and aesthetic try‐in, a single‐unit screw‐retained metal ceramic crown was delivered. The restorations, fabricated using a cobalt‐chromium‐based alloy, were directly connected to the implant collar. Prosthodontists had discretion to position the crown margin either at implant platform level or on the transmucosal collar, based on clinical judgment and specific case requirements. The prosthesis was inserted within two or 3 weeks after the impression. The fixation screw was tightened to 30 Ncm torque according to manufacturer's guidelines and the screw access was closed with light‐cured composite resin.

Patients were recalled every 6 months for hygiene maintenance and clinically checked for plaque and bleeding status.

### 2.4 Radiographic Measurements

**FIGURE 1:**
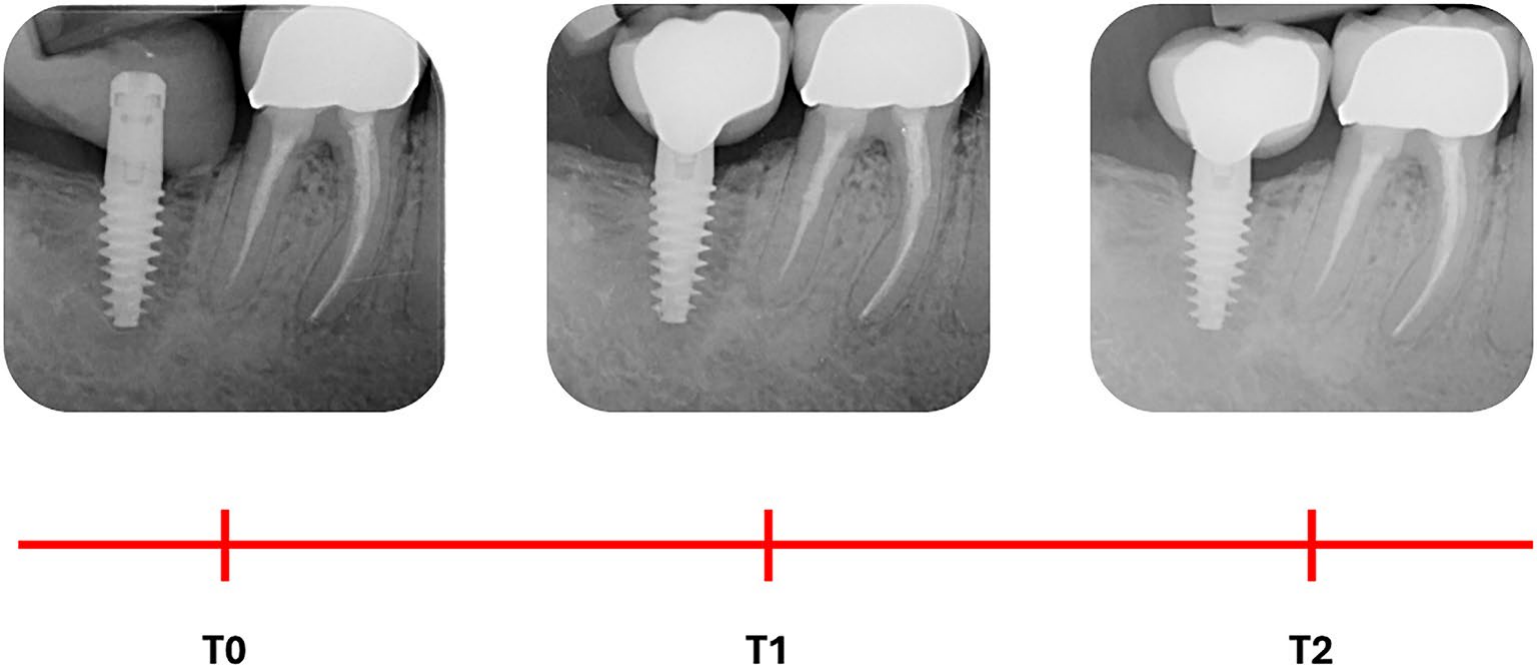
Illustrative case of a Group 3 (thick mucosa) implant: Periapical radiographs, customized with patient‐specific bite jig, were taken at implant placement (T0), at prosthesis delivery (T1), and after 18 months of functional loading (T2).

Digital radiographs were taken using a long‐cone paralleling technique with a Rinn‐type film holder, customized with patient‐specific bite jigs, at implant placement (baseline, T0), at crown delivery (T1) and after 18 months of prosthetic functional loading (T2) (Figure 1).

All radiographs were performed using the same X‐ray generator technology (FOCUS, KaVo, Biberach, Germany), set to the same parameters (60 kV, 7 mA).

Peri‐implant bone levels (PBL) were calculated at T1 and T2 on the mesial and distal sides of each implant as the linear measurement of the distance between two points: the most apical point of the crown margin and the most coronal bone‐to‐implant contact.

Measurements were corrected referring to the known height and diameter of each implant. An increased vertical distance between the most apical point of the crown margin and the most coronal bone‐to‐implant contact is considered indicative of bone resorption, while a decreased distance is considered indicative of bone gain.

PBL variations were subsequently categorized into two distinct components (BR and BL), as previously described [20, 35]. Specifically, Bone Remodeling (BR) refers to marginal bone changes occurring around the transmucosal portion of the implant (machined collar), without exposure of the roughened implant surface—typically observed when the smooth collar is partially positioned below the crestal bone level (Groups 1 and 2).

In contrast, BL is defined as marginal bone resorption apical to the machined collar, resulting in exposure of the rough implant surface (Groups 1, 2, and 3).

**FIGURE 2:**
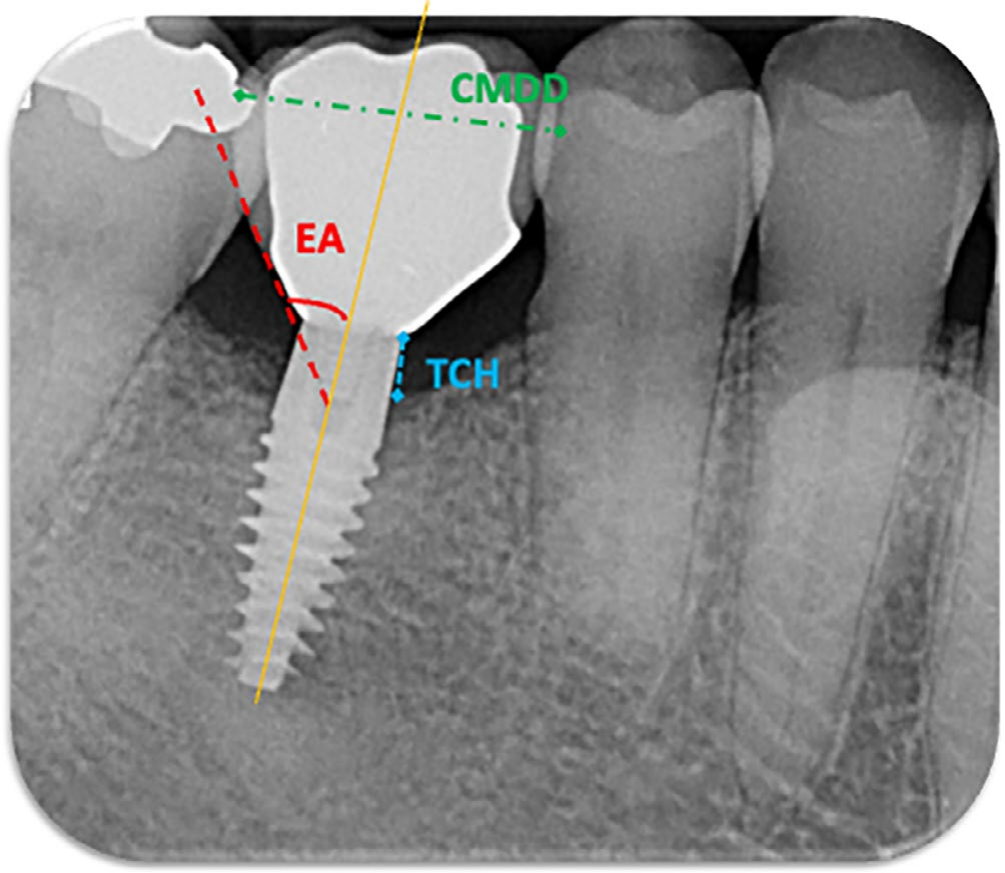
Crown Mesio‐Distal Dimension (CMDD) is the distance between the most mesial and the most distal point of the screwed crown (green dotted line). Emergence Angle (EA) is formed between the implant long axis (orange line) and a tangent line from the junction between the machined transgingival collar and the rough implant body, extending outward along the crown emergence profile (red dotted line). Transmucosal Collar Height (TCH) is the distance between the bone crest and the most apical point of the crown margin at mesial and distal aspects as measured at prosthesis delivery (yellow dotted line).

Measurement of the Primary Prosthetic Variables Was Performed at Each Implant After Crown Insertion, as Follows (Figure 2):

1. Crown Mesio‐Distal Dimension (CMDD): measured dicotomically (≥ 10 mm/< 10 mm) as the distance between the most mesial and the most distal point of the crown;
2. EA measured at mesial and distal sites by determining the angle between the implant long axis and a tangent line from the junction between the machined transgingival collar and the rough implant body, extending outward along the crown emergence profile;
3. Transmucosal Collar Height (TCH) measured dicotomically (> 2 mm/≤ 2 mm) as the distance between the bone crest and the most apical point of the crown margin at mesial and distal sites at prosthesis delivery (T1).

Radiographs demonstrating deformation, darkness, and/or other problems were immediately repeated. All measurements were made by two calibrated examiners, blind to mucosal thickness, using a 30‐in. LED‐backlit color diagnostic display with Kodak Digital Imaging Software (Kodak, Eastman Kodak, Rochester, NY, USA). Each measurement was performed three times at each of three different time points as proposed by Gomez‐Roman and Launer [36]. Examiner calibration was performed by measuring PBL on a sample of 10 radiographs not included in the study. Cohen's *k* coefficient for intra‐examiner and inter‐examiner agreement was 87.2% and 81.6%, respectively, for linear measurements within ±0.1 mm.

### 2.5 Statistical Analysis

Statistical analysis was conducted using IBM SPSS Statistics for Windows, version 26.0 (IBM Corp., Armonk, NY). Each patient, represented by one implant, served as the statistical unit. To identify significant differences across the three treatment groups, a sample size of 15 patients per group was determined necessary, with a confidence level of 5% and statistical power of 80%, assuming an anticipated difference in MBL of 0.3 mm (±0.25 mm) [37]. Descriptive statistics were presented as mean ± standard deviation for normally distributed data, and as median with interquartile range (IQR) for nonnormally distributed data. A paired‐samples t‐test was conducted to assess potential merging of mesial and distal measurements. Data normality was assessed using the Shapiro–Wilk test, and homogeneity of variances was evaluated using Levene's test. One‐way ANOVA was used to analyze the differences among groups, followed by Tukey's post hoc test to identify specific group differences. Multiple linear regression analysis was conducted using the enter method to evaluate the relationship between the dependent variable (MBL) and the independent variables (age, gender, smoking habits, history of periodontitis, vertical mucosal thickness, implant insertion torque, TCH, crown emergence profile, and mesio‐distal crown dimension). The null hypothesis (no significant association exists between marginal BR from T1 to T2 and the evaluated patient‐ or prosthetic‐related variables) was rejected at a critical significance level of *p* < 0.05.

## 3 Results

A total of 49 consecutively selected patients were enrolled and treated with the placement of 49 tissue‐level implants across six clinical centers (SS: 8 patients; FB: 8 patients; TL: 8 patients; CMS: 8 patients; MM: 8 patients; CS: 9 patients), as detailed in a previous study [20]. Following prosthesis delivery, two Group 1 patients did not attend follow‐up visits and were excluded from the study.

The final analysis included 47 implants in 47 patients (25 males and 22 females, mean age 58.3 ± 12.2 years, range 34–85 years) followed for 18 months after prosthetic loading (T2). One Group 2 implant failed after 9 months of functional loading. Forty‐six implants were successfully in function at T2, with no complications or adverse events recorded (2.1% implant failure rate after 18 months of loading). These were distributed as follows: Group 1: 16 implants; Group 2: 15 implants; Group 3: 15 implants. Complete demographic data are listed in Table 1.

**TABLE 1:** Demographic characteristics of the patients included in the final analysis.

	Thin mucosa (tot:16)	Medium mucosa (tot:16)	Thick mucosa (tot:15)
Age	59.3 ± 11.7	56.7 ± 12.2	59.2 ± 12.7
Gender (Male/Female)	10(62.5%)/6(37.5%)	6(37.5%)/10(62.5%)	9(60.0%)/6(40.0%)
Smoking habits (Yes/No)	6(37.5%)/10(62.5%)	2(12.5%)/14(87.5%)	4(26.7%)/11(73.3%)
History of periodontitis (Yes/No)	5(31.3%)/11(68.7%)	2(12.5%)/14(87.5%)	6(40.0%)/9(60.0%)

### 3.1 Radiographic Measurements

No significant differences were observed between mesial and distal PBL within any of the three groups at any time point. Therefore, a single PBL value, calculated as the mean of the mesial and distal measurements, was assigned to each implant.

From prosthesis delivery (T1) to 18 months post‐loading (T2), mean marginal bone resorption of the entire pool of patients was 0.14 ± 0.56 mm (0.41 ± 0.59 mm in Group 1 and 0.08 ± 0.56 mm in Group 2). In contrast, Group 3 showed an increase in PBLs, with a mean bone gain of 0.07 ± 0.43 mm. One‐way ANOVA followed by a post hoc Tukey HSD test revealed a significant difference from T1 to T2 between Group 1 and Group 3 (*p* = 0.041).

**FIGURE 3:**
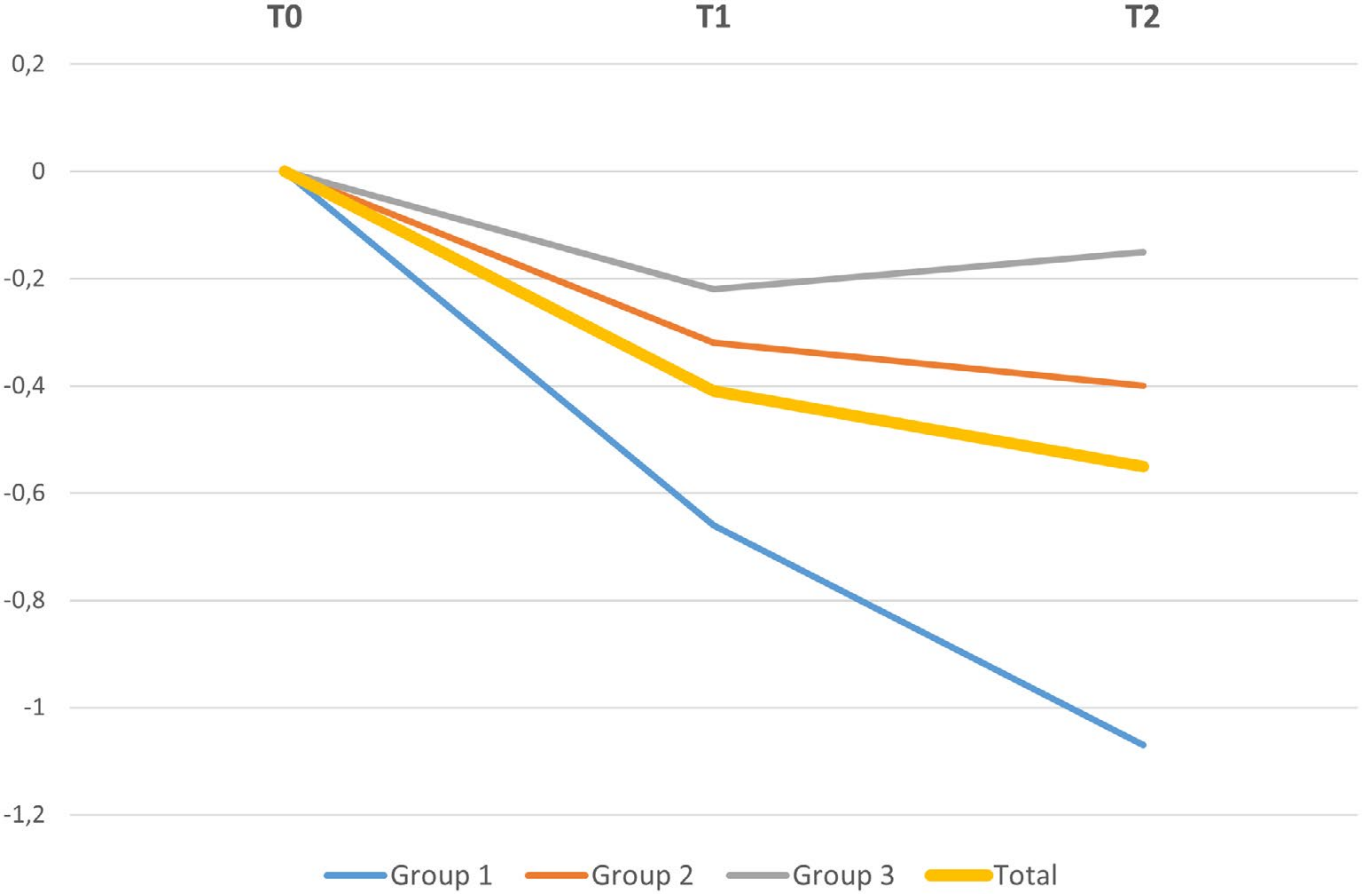
Marginal bone variations in the entire pool of patients and in the three groups (Group 1: thin mucosa; Group 2: medium mucosa; Group 3: thick mucosa) from baseline (T0) to prosthesis delivery (T1), up to 18 months of functional loading (T2).

Mean marginal bone resorption from T0 to T2 around all 46 implants included in the present investigation was 0.57 ± 0.72 mm (1.05 ± 0.58 mm in Group 1; 0.37 ± 0.64 mm in Group 2; and 0.25 ± 0.70 mm in Group 3) (Figure 3). One‐way ANOVA with post hoc Tukey HSD test indicated that bone resorption from T0 to T2 was significantly greater in Group 1 compared to Group 2 (*p* = 0.015) and Group 3 (*p* = 0.003).

However, when PBL variations at T2 are classified according to the criteria proposed by Spinato et al. [35] into BR and BL, a distinct distribution emerges among the groups, which appears to reflect differences in the apico‐coronal positioning of the implant at baseline, based on mucosal thickness. In Group 1 (thin mucosa), where implants were placed 2 mm subcrestally to compensate the limited soft tissue dimension, no cases of BL were observed; all 16 implants exhibited BR confined to the machined transmucosal portion. In Group 2 (medium mucosa, 1 mm subcrestal placement), only 1 of 15 implants exhibited BL, while 14 showed BR. In contrast, Group 3 (thick mucosa), where implants were placed at the crestal level, showed a higher incidence of BL (7 out of 15 implants).

Multivariate analysis revealed no significant associations between marginal bone changes from T1 to T2 and factors such as age, gender, smoking habits, history of periodontitis, vertical mucosal thickness, implant insertion torque, TCH, crown emergence profile, and mesio‐distal crown dimension. Detailed results can be found in Tables 2 and 3.

**TABLE 2:** Multiple linear regression analysis to assess the impact of independent variables on the dependent variable MBL (T1‐T2) on the mesial aspect of tissue‐level implants.

	Coefficient *B*	95% CI	*p*
Gender			
Male	1		
Female	−0.168	[−0.614 to 0.277]	0.447(ns)
Age	0.009	[−0.015 to 0.032]	0.460 (ns)
Smoking Habits			
Yes	1		
No	0.069	[−0.488 to 0.626]	0.801 (ns)
History of Periodontitis			
Yes	1		
No	−0.425	[−1.027 to 0.178]	0.161 (ns)
Implant Insertion Torque	0.004	[−0.011 to 0.018]	0.611 (ns)
Mucosal Thickness	−0.150	[−0.463 to 0.164]	0.338 (ns)
CMDD			
≥ 10 mm	1		
< 10 mm	0.125	[−0.408 to 0.657]	0.637 (ns)
TCH			
Long (> 2 mm)	1		
Short (≤ 2 mm)	0.012	[−0.464 to 0.489]	0.958 (ns)
EA mesial	−0.013	[−0.043 to 0.017]	0.369 (ns)

Abbreviations: CI: confidence interval; CMDD: Crown Mesio‐Distal Dimension; EA: emergence angle; ns: not statistically significant; TCH: transmucosal collar height.

**TABLE 3:** Multiple linear regression analysis to assess the impact of independent variables on the dependent variable MBL (T1‐T2) on the distal aspect of tissue‐level implants.

	Coefficient *B*	95% CI	*p*
Gender			
Male	1		
Female	−0.265	[−0.762 to 0.232]	0.286 (ns)
Age	0.014	[−0.010 to 0.038]	0.236 (ns)
Smoking Habits			
Yes	1		
No	0.064	[−0.497 to 0.625]	0.817 (ns)
History of Periodontitis			
Yes	1		
No	−0.500	[−1.108 to 0.107]	0.103 (ns)
Implant Insertion Torque	0.006	[−0.009 to 0.021]	0.454 (ns)
Mucosal Thickness	−0.176	[−0.484 to 0.131]	0.251 (ns)
CMDD			
≥ 10 mm	1		
< 10 mm	−0.094	[−0.657 to 0.469]	0.736 (ns)
TCH			
Long (> 2 mm)	1		
Short (≤ 2 mm)	0.009	[−0.510 to 0.493]	0.973 (ns)
EA distal	0.010	[−0.024 to 0.044]	0.607 (ns)

Abbreviations: CI: confidence interval; CMDD: Crown Mesio‐Distal Dimension; EA: emergence angle; ns: not statistically significant; TCH: transmucosal collar height.

However, the analysis did show a significant negative association between marginal bone resorption from T0 to T2 and vertical mucosal thickness (*p* = 0.007), as well as a significant positive association between marginal bone resorption from T0 to T2 and smoking habits (*p* = 0.028). No significant associations were found between marginal bone variations from T0 to T2 and other potential influencing factors, including age, gender, history of periodontitis, and insertion torque. The complete results of the multivariate analysis are presented in Table 4.

**TABLE 4:** Multiple linear regression analysis to assess the impact of independent variables on the dependent variable MBL from implant placement to 18 months of functional loading (T0‐T2).

	Coefficient *B*	95% CI	*p*
Gender			
Male	1		
Female	−0.102	[−0.551 to 0.347]	0.648 (ns)
Age	0.001	[−0.022 to 0.023]	0.942 (ns)
Smoking Habits			
Yes	1		
No	0.580	[0.067 to 1.092]	0.028 (s)
History of Periodontitis			
Yes	1		
No	0.010	[−0.596 to 0.617]	0.973 (ns)
Implant Insertion Torque	0.004	[−0.011 to 0.018]	0.626 (ns)
Mucosal Thickness	−0.365	[−0.623 to −0.106]	0.007 (s)

Abbreviations: CI: confidence interval; ns: not statistically significant; s: statistically significant.

## 4 Discussion

This prospective study investigates if prosthetic‐related (CMDD, EA, TCH) and host‐related (age, gender, history of periodontitis, smoking habits, mucosal thickness) factors influence MBL around tissue‐level implants from prosthesis delivery (T1) to 18 months of functional loading (T2). The present investigation is the continuation of a previous study in which the effect of STH adhesion on peri‐implant marginal bone level was evaluated prior to crown delivery and occlusal loading [20].

A histologic study in humans demonstrated that STH adhesion begins when implants are exposed to the oral cavity and is fully established within approximately 2 months [15]. Therefore, it can be assumed that peri‐implant STH adhesion around tissue‐level implants, along with any related marginal bone changes, is complete after the five‐month unsubmerged healing phase following implant placement [20]. This assumption is supported by the findings of the present study, which show that vertical mucosal thickness does not significantly affect PBLs from crown delivery (T1) to 18 months of functional loading (T2). These results align with other 12‐month clinical studies using tissue‐level implants with a different collar design [19, 38].

Although a limited number of studies have suggested that vertical mucosal thickness may influence EMBL around bone‐level implants even after prosthetic rehabilitation and during functional loading [39, 40], the majority of clinical evidence does not support this association. Several well‐designed trials have reported no significant impact of mucosal thickness on marginal bone levels, both in tissue‐level [19, 38] and bone‐level implant systems [21, 22, 28, 41, 42]. These findings suggest that the influence of mucosal thickness is primarily confined to the early healing period, specifically during the establishment of STH, which occurs prior to prosthesis delivery and functional loading [17, 18, 19, 20, 21]. Nonetheless, the overall impact of mucosal thickness should not be overlooked. In the present study, multivariate analysis revealed a significant negative association between thin mucosa and peri‐implant bone stability over the full observational period (T0–T2), suggesting that, even if bone changes predominantly occur during the early healing phase, their effect persists and contributes to the long‐term marginal bone condition.

Multivariate analysis also revealed that the examined prosthetic factors (CMDD, EA, and TCH) exert no significant influence on marginal bone levels from T1 to T2. Specifically, large crowns (with a mesio‐distal dimension ≥ 10 mm, such as molars) had the same effect on peri‐implant marginal BL as smaller crowns (with a mesio‐distal dimension < 10 mm, such as premolars). These findings are consistent with previous studies that demonstrated no significant correlation between occlusal table width and marginal bone stability [43, 44].

Recent pre‐clinical and clinical studies suggested that restoration emergence profile characteristics potentially influence marginal bone stability around bone‐level implants [30, 45, 46]. Over‐contoured emergence profiles appear to affect EMBL by hindering soft tissue adaptation and promoting plaque accumulation [47]. However, evidence linking prosthesis emergence profile to peri‐implant marginal bone stability remains limited [48, 49]. Findings in the present study indicate that crown EA does not influence marginal bone levels around tissue‐level implants, consistent with the results of Katafuchi et al. [30]. In their radiographic study, an EA greater than 30° was identified as a significant risk factor for peri‐implant BL in bone‐level implants but not in tissue‐level implants.

**FIGURE 4:**
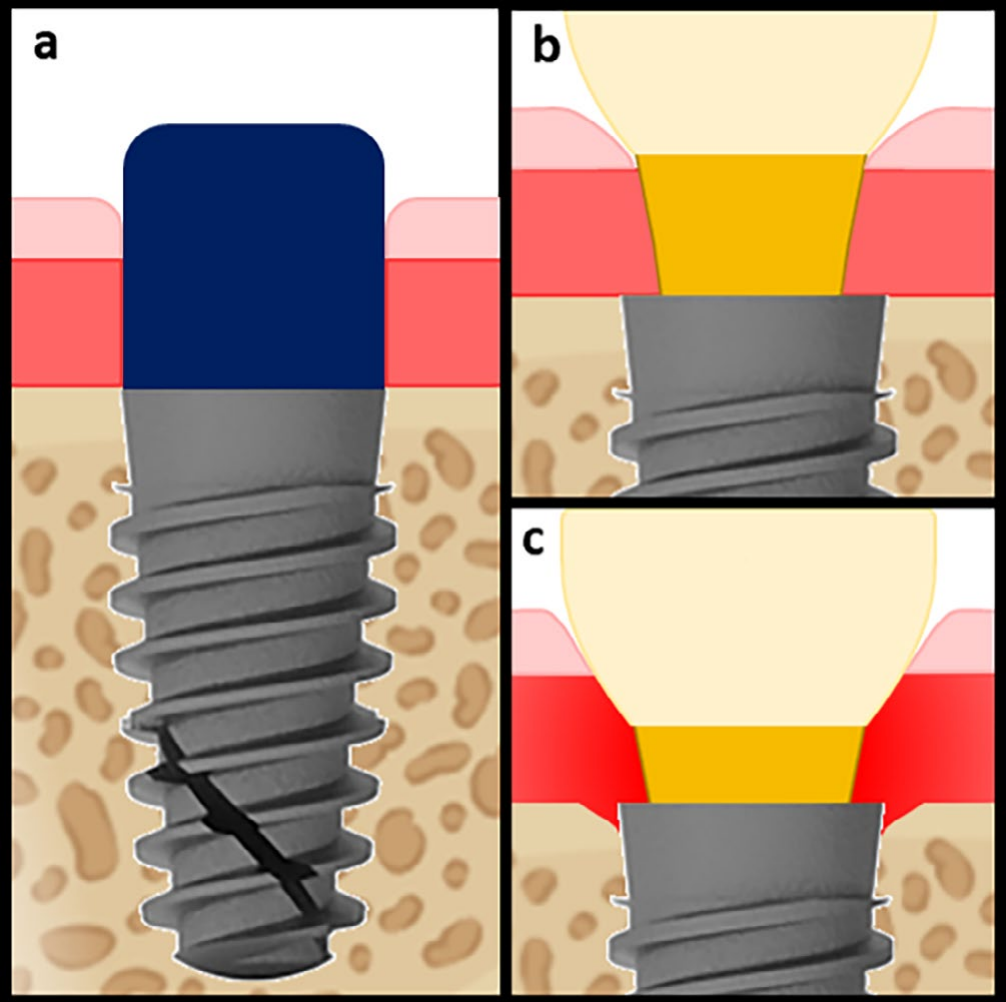
After soft tissue healing following bone‐level implant uncovering, the supracrestal tissue height is composed of the sulcus, the junctional epithelium, and a connective tissue portion (a). If the abutment is sufficiently long, the prosthetic crown margin will be located within the sulcus, without invading the underlying connective tissue space (b), thereby ensuring tissue stability. Conversely, if a short abutment is used, the prosthetic restoration will encroach upon the connective tissue zone, leading to inflammation and subsequent crestal bone resorption (c), which occurs to allow re‐establishment of an appropriate supracrestal tissue height in a more apical position.

**FIGURE 5:**
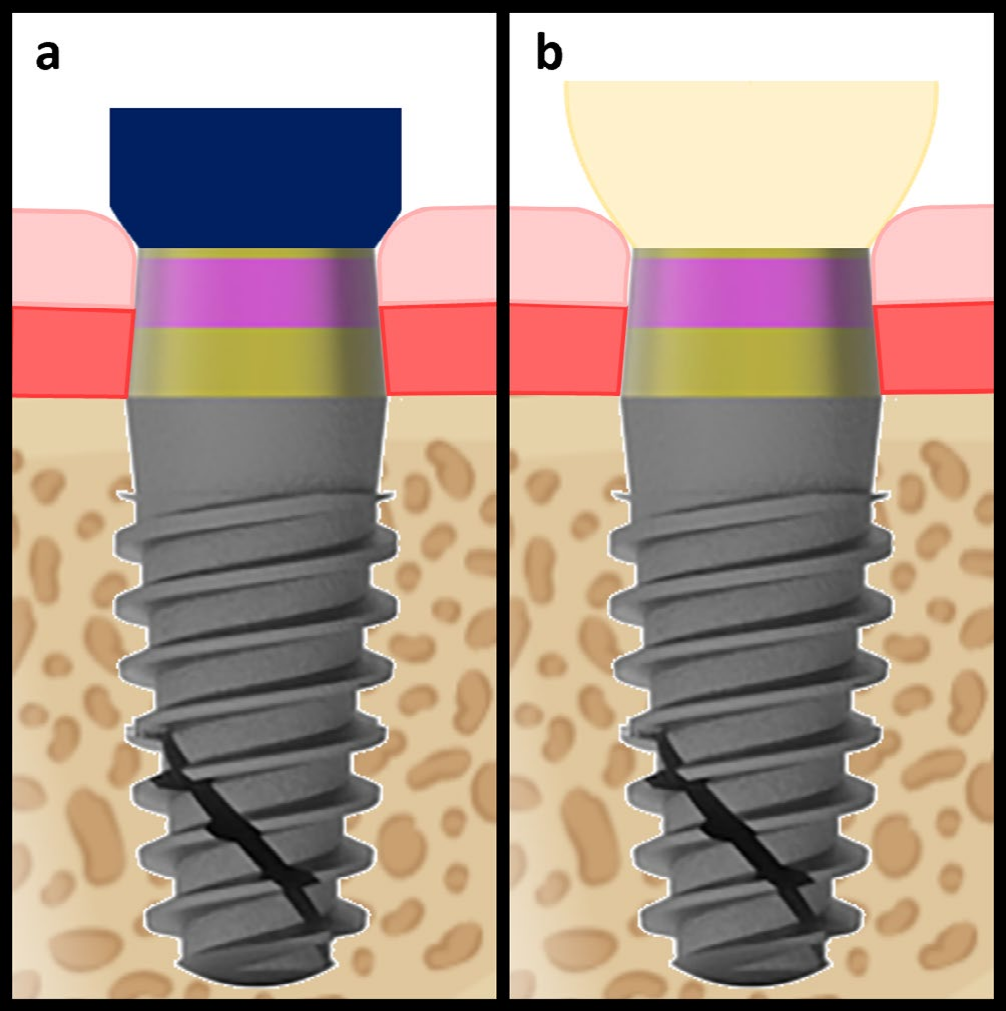
Following soft tissue healing after uncovering of a tissue‐level implant, the supracrestal tissue height exhibits an anatomical structure comparable to that surrounding bone‐level implants (a). However, during impression taking, the junctional epithelium and the connective tissue portion remain undisturbed by the removal of the healing abutment. This allows the dental technician to clearly visualize the contours of the healed sulcus and accurately position the prosthetic margin within it, without invading the underlying soft tissues (b).

TCH in tissue‐level implants is defined as the distance between the bone crest and the prosthetic crown margin at the time of delivery (T1). The results of the present study show no statistically significant correlation between TCH (high > 2 mm/low ≤ 2 mm) and marginal BL from T1 to T2. This finding contrasts with numerous studies on bone‐level implants that consistently associate prosthetic abutment height (high > 2 mm/low ≤ 2 mm) with marginal bone stability [21, 22, 24, 26, 27, 28, 29, 42]. This difference can be explained by the fact that the tissue‐level implants used in the present study represent a completely different scenario, and findings from bone‐level implants cannot be automatically applied to them. First, human studies have demonstrated that the average vertical dimension of STH is significantly lower in tissue‐level implants compared to bone‐level implants (2.55 ± 0.16 mm vs. 3.26 ± 0.15 mm) [50]. Additionally, other studies have reported even lower peri‐implant STH around tissue‐level implants with convergent transmucosal profiles (as used in the present study) compared to implants with straight transmucosal profiles [51, 52]. Moreover, and arguably of greatest relevance, the selection of abutment height—and thus the vertical position of the crown margin—in bone‐level implants is determined without precise knowledge of the individual connective tissue height and sulcus depth (Figure 4). Conversely, in tissue‐level implants, the impression accurately captures the transmucosal morphology, enabling the technician to define the emergence profile and crown margin within the gingival sulcus while respecting the integrity of the underlying connective tissue seal (Figure 5).

Other prosthetic parameters, such as crown‐to‐implant ratio, occlusal table width, or cusp height, were not included in the present analysis, as current literature does not support a direct association between these variables and marginal bone level changes. Therefore, only prosthetic factors previously suggested as potential influencers of marginal bone stability—namely, EA, mesio‐distal crown dimension, and TCH—were selected for investigation in this study.

The present study finds that smoking negatively affects healing and peri‐implant marginal bone stability from T0 to T2, as well demonstrated in previous clinical trials and systematic reviews [6, 7, 53, 54]. Detrimental effects of smoking include impaired wound healing, reduced collagen production, compromised fibroblast function, impaired peripheral circulation, and diminished phagocytic activity. Consequently, local exposure of peri‐implant tissues to tobacco products negatively influences marginal bone stability and implant survival [55]. A recent systematic review and meta‐analysis investigating the relationship between smoking levels and MBL revealed that smokers experience significantly greater MBL compared to non‐smokers. Additionally, smokers consuming more than 10 cigarettes per day showed significantly higher MBL compared to those smoking less than 10 cigarettes per day, highlighting a dose‐dependent increase in MBL with higher smoking levels [56].

When PBL variations are classified into BR and BL, the T2 results reveal a clear pattern associated with mucosal thickness. In Group 1 (thin mucosa), no cases of BL were observed; all 16 implants exhibited only BR. In Group 2 (medium mucosa), BL was noted in 1 of 15 implants, while the remaining 14 showed BR. In contrast, Group 3 (thick mucosa) demonstrated a higher incidence of BL, with 7 out of 15 implants affected, and only 8 exhibiting BR. These findings suggest that proactively adapting the apico‐coronal positioning of the implant based on mucosal thickness—thereby anticipating the vertical dimension required for supracrestal tissue attachment—may reduce the risk of exposure of the roughened implant surface 18 months after prosthetic loading. Based on this evidence, positioning the machined collar approximately 0.5–1 mm below the bone crest even in cases of thick mucosa may represent a prudent strategy to minimize the occurrence of BL.

The present study has several limitations that should be considered when interpreting the findings. First, the sample size, although adequate for statistical analysis, remains relatively small and focused on a specific anatomical region (the posterior mandible). This limits the generalizability of the results to other clinical scenarios, such as the maxilla or anterior regions, where different biomechanical and soft tissue conditions may apply. Second, the observational period of 18 months post‐loading, while sufficient to assess mid‐term marginal bone changes, does not provide information on long‐term peri‐implant bone stability or the risk of late complications, such as peri‐implantitis. Third, radiographic evaluation was based exclusively on standardized two‐dimensional periapical radiographs. While this method is commonly used and clinically practical, it does not allow for assessment of buccal and lingual bone levels, which may also be affected by the investigated variables. Similarly, it does not provide complete characterization of the emergence profile or crown morphology in three dimensions. Fourth, although multiple variables were included in the multivariate analysis, other potentially relevant prosthetic factors—such as crown‐to‐implant ratio, occlusal table width, and cusp inclination—were not analyzed due to the absence of validated evidence suggesting their association with marginal bone changes, as well as limitations in data standardization across multiple centers.

Finally, despite efforts to standardize procedures through a calibration meeting and protocol harmonization, the multicenter nature of the study inherently introduces operator variability in clinical execution, radiographic technique, and prosthetic design choices. Although this reflects real‐world practice and enhances external validity, it may also increase variability in the measurements.

Future studies involving larger, more diverse populations and three‐dimensional imaging techniques (e.g., CBCT) are recommended to validate and expand upon these findings.

## 5 Conclusion

From prosthesis delivery (T1) to 18 months post‐loading (T2), PBL remained relatively stable, with no significant differences between the three groups. Multivariate analysis revealed no significant associations between PBL changes and patient demographics or prosthetic factors.

Mean total marginal bone resorption from implant placement (T0) to T2 was significantly higher in smokers and in implants with thin mucosa (Group 1) compared to those with medium (Group 2) and thick mucosa (Group 3). The present findings emphasize the relationship between thin mucosa and greater peri‐implant bone resorption, particularly during the unsubmerged healing phase prior to prosthesis delivery (T0‐T1), and underline the detrimental impact of smoking on peri‐implant bone stability.

## Author Contributions

**Sergio Spinato:** concept/design, data collection, data analysis/interpretation, article drafting, article approval. **Fabio Bernardello:** data collection, critical revision of article, article approval. **Claudio Stacchi:** data collection, data analysis/interpretation, article drafting, article approval. **Carlo Maria Soardi:** data collection, critical revision of article, article approval. **Marcello Messina:** data collection, critical revision of article, article approval. **Antonio Rapani:** statistics, critical revision of article, article approval. **Teresa Lombardi:** concept/design, data collection, critical revision of article, article approval.

## Conflicts of Interest

The authors declare no conflicts of interest.

## Data Availability

The data that support the findings of this study are available on request from the corresponding author. The data are not publicly available due to privacy or ethical restrictions.
